# Editorial: Advances in targeted therapy and biomarker research for endocrine-related cancers

**DOI:** 10.3389/fendo.2024.1533623

**Published:** 2024-12-11

**Authors:** Yang Wu, Zili Zhang

**Affiliations:** ^1^ School of Pharmacy, Nanjing University of Chinese Medicine, Nanjing, China; ^2^ Pancreas Center, the First Affiliated Hospital of Nanjing Medical University, Nanjing, China

**Keywords:** targeted therapy, biomarker, endocrine-related cancers, molecular mechanisms, clinical applications

The field of endocrine-related cancers is undergoing rapid transformation, fueled by significant strides in deciphering the molecular and genetic foundations of these diseases (1). These cancers, which encompass a range of malignancies affecting the endocrine system, present unique challenges due to their intricate interactions with hormonal signaling pathways (2). In recent years, the surge in the development of targeted therapies has sparked optimism for more precise and personalized treatment options (3). Simultaneously, the identification and validation of biomarkers have become crucial for improving diagnosis, prognosis, and treatment decision-making, marking a significant shift toward precision medicine. Moreover, cutting-edge research techniques, including organoid cultures and organ-on-a-chip systems, have emerged, providing more accurate and reliable models for studying tumor biology and drug responses (4). These innovations highlight the growing importance of translational medicine, which bridges the gap between laboratory findings and clinical applications, opening new pathways for understanding and addressing endocrine-related cancers. This dynamic landscape calls for a comprehensive examination of both current trends and future directions in the research and treatment of these complex diseases.

The aim of this Research Topic was to accelerate pioneering research and deepen our understanding of endocrine-related cancers. By spotlighting groundbreaking studies on targeted therapies, biomarker discovery, and advanced modeling approaches like organoids and organ-on-a-chip platforms, we seek to foster a connection between experimental research and clinical practice. Our goal was to establish a collaborative forum that not only disseminates cutting-edge scientific insights but also stimulates the development of innovative therapeutic strategies, ultimately enhancing patient outcomes in the field of endocrine oncology. A series of articles have been published under this Research Topic, as shown below.

Head and neck paragangliomas (HNPGNs) are infrequent tumors that originate from the parasympathetic paraganglia located at the skull base and neck (5). Standard treatment options, including surgery and radiotherapy, carry considerable risks due to the tumors’ close proximity to critical blood vessels and cranial nerves (6). Diagnosing HNPGNs is particularly challenging, as distinguishing between benign and malignant forms cannot be solely achieved through histology or imaging (7). Gaining a deeper understanding of the tumor microenvironment in HNPGNs could unveil key markers associated with tumor growth and malignancy, potentially enabling earlier detection, more effective targeted therapies, and strategies to prevent recurrences and metastasis. Therefore, Baruah et al. examined the profiles of fibroblasts and macrophages within HNPGNs. They identified the expression of fibroblast markers CD90 and PDPN in HNPGN tissue *in-situ* as well as presence of CD163 expressing macrophages. By isolating fibroblasts from HNPGN tissue, they confirmed CD90 expression *in vitro* and further detected monocarboxylate transporters (MCT1) in HNPGN-derived fibroblasts through flow cytometry. They also observed MCT1 and MCT4 expression in both tumor cells and macrophages within the HNPGN tissue. Further investigation of the phenotypic and functional characteristics of fibroblasts and macrophages in HNPGNs is crucial for better understanding the tumor microenvironment, which could lead to novel approaches for risk stratification and the development of targeted therapies.

Breast cancer is a leading global malignancy in women, with increasing incidence rates (8). It significantly impacts patients’ quality of life and health. Despite treatment advancements, challenges persist, including delayed diagnosis, unpredictable outcomes, and drug resistance (9). Consequently, identifying new biomarkers and therapeutic targets is a key focus of current breast cancer research. Therefore, Song et al. conducted a comprehensive proteome-wide Mendelian randomization (MR) study to uncover potential biomarkers and therapeutic targets for breast cancer. Their study identified several proteins linked to increased breast cancer risk, including decreased levels of CASP8, DDX58, CPNE1, ULK3, PARK7, and BTN2A1, as well as elevated levels of TNFRSF9, TNXB, DNPH1, and TLR1. Among these, CASP8 and DDX58 were supported by tier-one evidence, while CPNE1, ULK3, PARK7, and TNFRSF9 received tier-two evidence support. The remaining proteins, TNXB, BTN2A1, DNPH1, and TLR1, were supported by tier-three evidence. Notably, several of these proteins, such as CASP8, DDX58, CPNE1, ULK3, PARK7, and TNFRSF9, are already recognized as potential drug targets. Moving forward, integrating multi-omics data, including expression quantitative trait loci (eQTL) and methylation quantitative trait loci (mQTL), alongside MR and two-sample MR approaches, could offer deeper insights into the molecular mechanisms of breast cancer and aid in the identification of personalized therapeutic targets.

Thyroid cancer originates in the thyroid gland, a butterfly-shaped organ located at the base of the neck, and its incidence has been rising in recent years (10). Key risk factors include radiation exposure, genetic mutations, and family history, with women being more frequently affected (11). Treatment options are largely determined by the cancer’s type and stage, with surgery to remove the thyroid being a common approach. Remaining cancer cells are often treated with radioactive iodine therapy (12). Given the complexity of the disease and the need for personalized care, targeted therapies and biomarker research have become crucial areas of focus in thyroid cancer management. Guo et al. conducted a thorough review of conventional treatment strategies, the status of biomarker research, and the latest advancements in targeted therapy for thyroid cancer. They identified several critical molecular markers, including BRAF mutations, RAS mutations, RET/PTC rearrangements, PAX8/PPARγ rearrangements, and TERT promoter mutations, which are strongly associated with the disease. Understanding these biomarkers enables more precise diagnosis, prognostication, and tailored treatment plans for patients. Targeted treatments for thyroid cancer aim to disrupt the cancer cells’ growth mechanisms by targeting specific molecules involved in tumor development. Among the key approaches identified are tyrosine kinase inhibitors, thyroid hormone receptor antagonists, radioactive iodine therapy, immunotherapy, and gene-targeted therapies. Further investigation is focused on discovering new therapeutic targets, improving treatment tolerance, and developing combination therapies. The evolving scientific landscape provides hope for more effective, personalized treatment options for thyroid cancer.

Liver hepatocellular carcinoma (LIHC) is the most prevalent primary liver cancer and the third leading cause of cancer-related deaths worldwide (13). It affects individuals with chronic liver diseases, such as those caused by viral hepatitis, alcohol-related liver damage, or non-alcoholic fatty liver disease (14). Identifying clinical and biochemical factors that can pinpoint high-risk groups is crucial for timely intervention through imaging or screening. Improving prevention efforts and developing novel therapies are essential for better outcomes. Yu et al. evaluated the causal impact of exposure factors, including Alzheimer’s disease, platelet count, ambidextrousness, cigarettes smoked per day, alcohol consumption, and endocarditis, on the risk of LIHC using a two-sample MR study. Their findings provided compelling evidence supporting a causal relationship between reduced platelet levels and heightened vulnerability to LIHC in the European population. Therefore, it is recommended to prioritize the management of individuals with lower platelet counts to minimize their risk of developing LIHC. As a result, this study contributes to an expanding body of literature indicating that targeting platelet-related agents holds promise as a potential therapeutic approach for early detection and treatment of LIHC.

In summary, the articles collected under this Research Topic provide a comprehensive examination of targeted therapies and biomarkers for endocrine-related cancers. By focusing on the molecular and genetic underpinnings of these tumors, the studies presented offer critical insights into improving diagnosis, prognosis, and treatment personalization. The identification and validation of specific biomarkers have the potential to enhance early detection and guide more precise, individualized treatment approaches. The development of targeted therapies, aimed at disrupting key signaling pathways involved in tumor growth, represents a promising avenue for improving patient outcomes. These findings contribute to shaping the future direction of clinical management for endocrine-related cancers. Lastly, we thank all our contributors who enriched this Research Topic by submitting manuscripts highlighting their highly valuable and interesting research studies.
